# Supplementary material for: Dexamethasone impairs the expression of antimicrobial mediators in lipopolysaccharide-activated primary macrophages by inhibiting both expression and function of interferon β
Source: Front Immunol. 2023 Oct 24;14:1190261. doi: 10.3389/fimmu.2023.1190261 (PMC10628473; doi:10.3389/fimmu.2023.1190261)
Supplement: Supplementary file 1 [file Image_1.pdf]

Figure S1

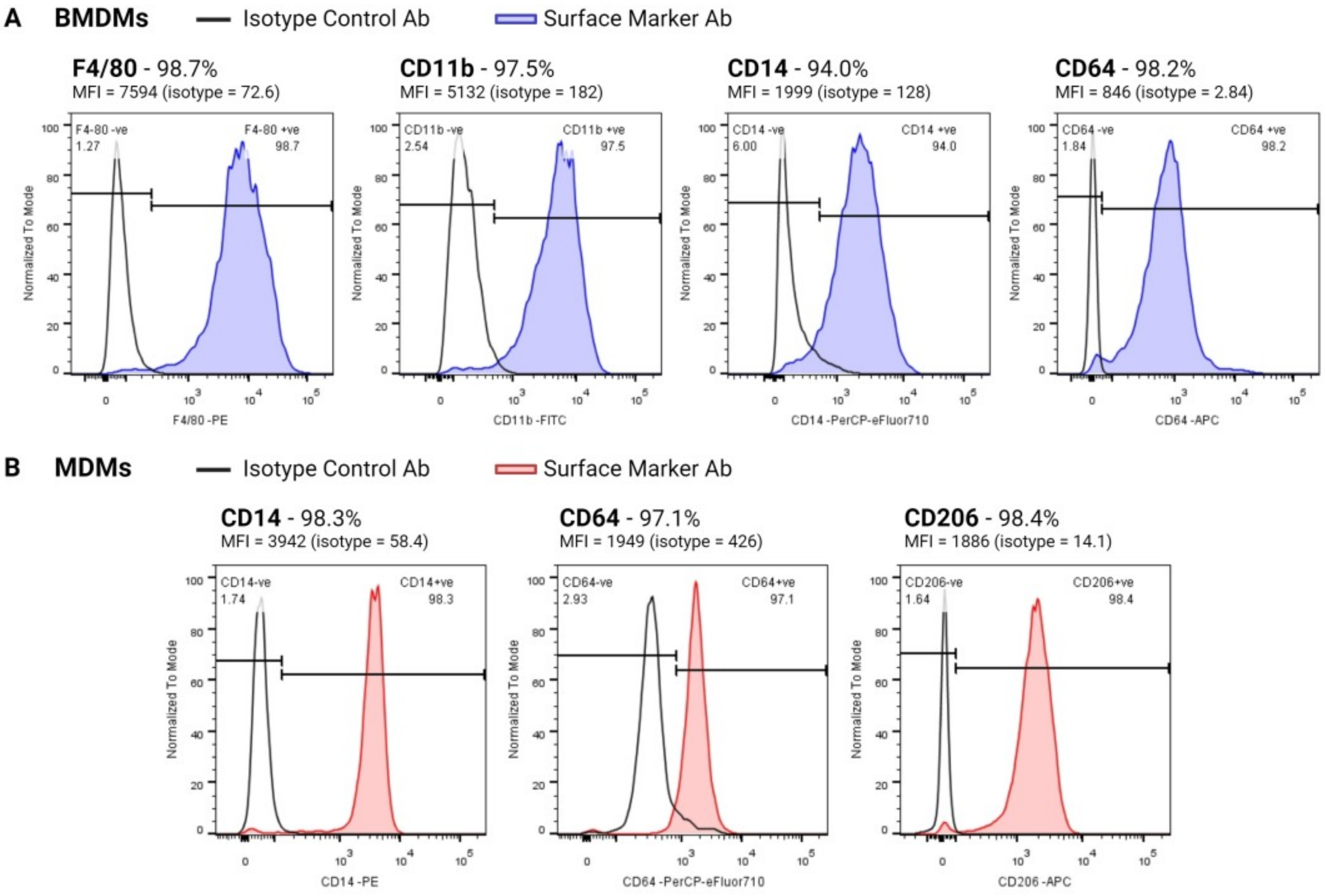

Figure S1. Quality control of primary macrophage populations. Macrophages were generated from mouse bone marrow **A)** or healthy human donor blood cones **B)** and stained with antibodies against myeloid or mature macrophage cell surface markers as described in Materials and Methods. Appropriate isotype control antibodies were used in each case. Representative flow plots are shown.
