# Supplementary material for: Dexamethasone impairs the expression of antimicrobial mediators in lipopolysaccharide-activated primary macrophages by inhibiting both expression and function of interferon β
Source: Front Immunol. 2023 Oct 24;14:1190261. doi: 10.3389/fimmu.2023.1190261 (PMC10628473; doi:10.3389/fimmu.2023.1190261)
Supplement: Supplementary file 2 [file Table_1.docx]

|  | **Term** | **GO** | **Log2Enrichment** | **-Log10FDR** | **Gene number** | **Gene ratio** | **Gene list** |
| --- | --- | --- | --- | --- | --- | --- | --- |
| 1 | cell activation | 0001775 | 2.669 | 10.294 | 26 | 0.043 | *Zbtb32, Lta, Ccl2, Rsad2, Arid5a, Il18, Ptgs2, Il1a, Csf2, Tnfsf15, Zfp36, Ripk2, Tnsf4, Cxcl5, Il4ra, Irf1, Il12b, Ccr7, Ifi202b, Dll1, Socs1, Gbp4, Il6, Cd200, Il10, Nod1, Il33, Il1f6, Lag3,* ***Ifnb1****, Il1b, Il1f9, il27* |
| 2 | regulation of cytokine production | 0001817 | 2.7866 | 14.387 | 33 | 0.047 | *Lta, Ccl2, Cxcl10, Ccl24, Il18, Ptgs2, Il1a, Cxcl3, Ccl8, Ccl12, Ccl7, Il2ra, Tnfsf4, Ccl22, Cxcl5, Il4ra, Olr1, Ccr7, Il6, Il1rn, Il1f6, Il1b, Cxcl9, Il1f9, Il27* |
| 3 | regulation of response to biotic stimulus | 0002831 | 2.852 | 5.7375 | 14 | 0.049 | *Ccl2, Tnip3, Cxcl10, Arid5a, Il18, Il1a, Zfp36, Cxcl3, Ccl12, Ripk2, Tnfsf4, Cxcl5, Cd86, Il12b, Il6, Cmpk2, Il10, Il1f6, Il1b, Cxcl9, Il1f9* |
| 4 | regulation of cell-cell adhesion | 0022407 | 2.9069 | 9.2441 | 21 | 0.051 | *H2-M2, Lta, Ccl2, Rsad2, Arid5a, Il18, Ripk2, Tnfsf4, Ahr, Cxcl5, Il4ra, Cd86, Irf1, Il12b, Cr7, Il6, Fgl2, Tnfsf18, Il10, Il33, Lag3,* ***Ifnb1****, Il1b, Il27* |
| 5 | regulation of immune effector process | 0002697 | 3.0583 | 11.623 | 24 | 0.057 | *Batf2, Gbp11, Il4ra, Gbp4, Il12b, Gbp4, Il6, Iigp1, Gbp9, Il10, Ier3* |
| 6 | inflammatory response | 0006954 | 3.0858 | 13.419 | 27 | 0.058 | *Zbtb32, Batf2, Rsad2, Cxcl10, Slamf7, Il18, Csf2, Hsh2d, Lck, Ripk2, Tnfsf4, Ahr, Cd86, Irf1, Tnfsf8, Il12b, Ccr7, Dll1, Il6, Cd70, Fgl2, Tnfsf18, Il33, Lag3,* ***Ifnb1****, Il1b* |
| 7 | negative regulation of cell activation | 0050866 | 3.1953 | 6.3116 | 13 | 0.063 | *Ccl2, Il18, Il1a, Slfn1, Il2ra, Lck, Ripk2, Tnfsf4, Il4ra, Cd86, Irf1, Il12b, Ccr7, Socs1, Il6, Fgl2, Il1rn, Tnfsf18, Il10, Lag3,* ***Ifnb1****, Il1b* |
| 8 | regulation of mononuclear cell proliferation | 0032944 | 3.2615 | 7.6536 | 15 | 0.066 | *Gm4841, Ifit3, Tgtp2, Irf1, Gbp4, Ifi202b, Iigp1, BC094916,* ***Ifnb1****, Tgtp1* |
| 9 | response to virus | 0009615 | 3.3813 | 7.762 | 16 | 0.061 | *Il18, Il1a, Slfn1, Il2ra, Ripk2, Tnfsf4, Ahr, Cd86, Irf1, Il12b, Ccr7, Il6, Tnfsf18, Il10, Il1b, Il27* |
| 10 | cellular response to biotic stimulus | 0071216 | 3.6803 | 13.195 | 21 | 0.088 | *Rsad2, Il18, Tnfsf4, Il4ra, Irf1, Il6, Il33,* ***Ifnb1*** |
| 11 | cellular response to interleukin-1 | 0071347 | 4.3067 | 7.3316 | 10 | 0.135 | *Ccl2, Ccl24, Il1a, Ccl8, Ccl12, Ccl7, Ccl22, Irf1, Il6, Il1b* |
| 12 | cellular response to interferon-beta | 0035458 | 4.9928 | 8.8633 | 10 | 0.217 | *Slfn1, Il2ra, Tnfsf4, Il4ra, Cd86, Irf1, Socs1, Cd200, Fgl2, Tnfsf18, Il10, Lag3,* ***Ifnb1*** |
| 13 | regulation of type 2 immune response | 0002828 | 5.2403 | 7.4841 | 8 | 0.258 | *Ripk2, Ahr, Cxcl5, Irf1, Il12b, Ifi202b, Socs1, Gbp4, Fgl2, Nod1, Lag3, BC094916, Il1b, Il27* |
| 14 | response to protozoan | 0001562 | 5.2965 | 10.644 | 11 | 0.268 | *Rsad2, Mx1, Cxcl10, Ifit2, Mx2, Tnfsf4, Ifit3, Cd86, Irf1, Il12b, Il6, Isg20, Il33,* ***Ifnb1****, Cxcl9, Tgtp1* |

**Supplemental Table 1.** Top 14 GO terms significantly enriched amongst LPS-induced and Dex-inhibited genes in mouse bone marrow-derived macrophages. Relates to Fig. 1B.
